# New insights on the role of human leukocyte antigen complex in primary biliary cholangitis

**DOI:** 10.3389/fimmu.2022.975115

**Published:** 2022-08-31

**Authors:** Giacomo Mulinacci, Andrea Palermo, Alessio Gerussi, Rosanna Asselta, Merrill Eric Gershwin, Pietro Invernizzi

**Affiliations:** ^1^ Division of Gastroenterology, Center for Autoimmune Liver Diseases, Department of Medicine and Surgery, University of Milano-Bicocca, Monza, Italy; ^2^ European Reference Network on Hepatological Diseases (ERN RARE-LIVER), San Gerardo Hospital, Monza, Italy; ^3^ Department of Biomedical Sciences, Istituti di Ricovero e Cura a Carattere Scientifico (IRCCS) Humanitas Research Hospital, Milan, Italy; ^4^ Department of Biomedical Sciences, Humanitas University, Milan, Italy; ^5^ Division of Rheumatology, Allergy and Clinical Immunology, University of California, Davis, Davis, CA, United States

**Keywords:** primary biliary cholangitis, human leukocyte antigens complex, HLA haplotypes, genetics, immunotolerance

## Abstract

Primary Biliary Cholangitis (PBC) is a rare autoimmune cholangiopathy. Genetic studies have shown that the strongest statistical association with PBC has been mapped in the human leukocyte antigen (HLA) locus, a highly polymorphic area that mostly contribute to the genetic variance of the disease. Furthermore, PBC presents high variability throughout different population groups, which may explain the different geoepidemiology of the disease. A major role in defining HLA genetic contribution has been given by genome-wide association studies (GWAS) studies; more recently, new technologies have been developed to allow a deeper understanding. The study of the altered peptides transcribed by genetic alterations also allowed the development of novel therapeutic strategies in the context of immunotolerance. This review summarizes what is known about the immunogenetics of PBC with a focus on the HLA locus, the different distribution of HLA alleles worldwide, and how HLA modifications are associated with the pathogenesis of PBC. Novel therapeutic strategies are also outlined.

## Introduction

Primary biliary cholangitis (PBC) is a rare pathology that can evolve to cirrhosis and liver failure. Although the etiology has not been completely explained, there is evidence supporting that autoimmunity targeting intrahepatic biliary ducts plays a central role in the pathogenesis (1).

PBC presents features that makes it a typical autoimmune condition. Firstly, it is characterized by the presence of specific autoantibodies, i.e. antimitochondrial autoantibodies (AMAs), which have a central role in diagnosis; secondly, the intense infiltrations of lymphocytes damaging bile ducts is a typical histological finding; finally, PBC patients have a high prevalence of concurrent autoimmune disorders (2). Like other autoimmune diseases, environmental and genetic factors have been called in action to explain its pathogenesis. PBC onset is thought to follow the interaction of an environmental trigger(s) with a predisposing genetic background. Concerning environmental factors, recurrent urinary tract infections, cigarette smoking, hair dyes, and the use of hormone replacement therapies have all been associated with increased risk of PBC (3). Furthermore, a growing number of studies have reported a higher prevalence in urban and polluted areas (4).

Genetic predisposition has a significant role in the onset of PBC too (5). The observation of clustering of cases within families and the high concordance rate between monozygotic twins support this claim. Örnolfsson et al. used a nationwide genealogical database and defined the relative risk for first-, second-, and third-degree relatives of PBC patients as 9.13, 3.16, and 2.59, respectively (6). The odds ratio for PBC of an individual with a sibling affected by the same condition is 10.5, in line with other autoimmune disorders (7). Further, the concordance rate for PBC in monozygotic twins is estimated to be 63%, among the highest reported in autoimmune conditions (3). From a genetic perspective, PBC is a complex trait, defined by Lander et al. as “any phenotype that does not exhibit classic Mendelian recessive or dominant inheritance attributable to a single gene locus” (8). This means that multiple gene variants increase or decrease the genetic risk with a relatively small effect size (9). A strong contribution in the advancement of the knowledge of PBC and its genetics has been given by the genome-wide association studies (GWAS). GWAS employ a large scanning of the entire genome for specific genetic variants (typically single nucleotide polymorphisms, SNPs) to identify alleles statistically associated with the disease (10). The strongest statistical association with PBC has been mapped in the human leukocyte antigen (HLA) locus. The HLA region is a highly polymorphic area of the genome located on chromosome 6p21 covering 6.7 Mb (11), and encoding hundreds of loci related to adaptive immune response, including histone and tRNA genes, several key immune response genes, as well as those of the major histocompatibility complex (MHC). The extended MHC region can be subdivided into three classes: class I (extended and classical, respectively, containing the *A, B, C* as well as *MICA* and *MICB* loci); class II (extended and classical, respectively, containing the *DPA1/DPB* and *DQA1/DQB1* loci); class III (containing the *DRA1/DRB1* loci).

Apart from the HLA region, a significant number of non-HLA genes have been identified, and many of the alleles involved genes implicated in innate immunity. In particular, studies have found alterations in genes and pathways involved in antigen presentation and production of interleukin (IL)-12 (*IRF5, SOCS1, TNFAIP3, NF-κB*, and *IL-12A*), activation of T cells, and interferon γ (IFN-γ) production (*TNFSF15, IL12R, TYK2, STAT4, SOCS1, NF-κB*, and *TNFAIP3*), as well as activation of B cells and production of immunoglobulins (*POU2AF1, SPIB, PRKCB, IKZF3*, and *ARID3A*) (**Table 1**) (12–20).

**Table 1:** List of PBC-related non-HLA associations.

*Chr*	*Locus*	*Candidate gene(s)*	*SNP*	*OR*	*Country*	*Reference*
*1*	1p13	CD58	rs2300747	1.30	Japan/China	*Qui et al.*
	1p31	IL12RB2	rs72678531	1.51	Europe/North America	*Liu et al.*
	1p36	MMEL1	rs3748816	1.33	Europe/North America	*Hirschfield et al.*
	1q31	DENND1B	rs12134279	1.34	Europe/North America	*Mells et al.*
*2*	2q12	IL1RL1, IL1RL2	rs12712133	1.14	Europe/North America	*Cordell et al.*
	2q32	STAT4, STAT1	rs10931468	1.50	Europe/North America	*Mells et al.*
	2q32	STAT4, STAT1	rs10168266	1.31	Japan/China	*Qui et al.*
	2q33	CD28, CRLA4, ICOS	rs4675369	1.37	Japan/China	*Qui et al.*
	2q33	CD28, CRLA4, ICOS	rs7599230	1.37	Japan/China	*Qui et al.*
	2q36	CCL20	rs4973341	1.22	Europe/North America	*Cordell et al.*
*3*	3p24	PLCL2	rs1372072	1.20	Europe/North America	*Mells et al.*
	3q13	CD80	rs2293370	1.39	Europe/North America/Japan/China	*Liu et al.*
	3q25	IL12A	rs2366643	1.35	Europe/North America/Japan/China	*Liu et al.*
*4*	4p16	DGKQ	rs11724804	1.22	Europe/North America	*Cordell et al.*
	4q24	NFkB1	rs7665090	1.26	Europe/North America/Japan/China	*Mells et al.*
*5*	5p13	IL7R	rs6871748	1.30	Europe/North America/Japan/China	*Liu et al.*
	5p13	IL7R	rs6897932	1.52	Europe/North America/Japan/China	*Kawashima et al.*
	5q21	C5orf30	rs526231	1.15	Europe/North America	*Cordell et al.*
	5q33	IL12B, LOC285626	rs2546890	1.15	Europe/North America	*Cordell et al.*
*6*	6q23	OLIG3, TNFAIP3	rs6933404	1.18	Europe/North America	*Cordell et al.*
*7*	7p14	ELMO1	rs6974491	1.25	Europe/North America	*Mells et al.*
	7q32	IRF5	rs35188261	1.52	Europe/North America	*Liu et al.*
*9*	9p32	TNFSF15	rs4979462	1.57	Japan/China	*Nakamura et al., Kawashima et al.*
	9p32	TNFSF15, TNFSF8	rs4979467	1.40	Japan/China	*Qui et al.*
*11*	11q13	RPS6KA4	rs538147	1.23	Europe/North America	*Mells et al.*
	11q23	POU2AF1	rs4938534	1.38	Japan/China	*Nakamura et al., Kawashima et al.*
	11q23	CXCR5, DDX6	rs77871618	1.55	Japan/China	*Qui et al.*
	11q23	CXCR5, DDX6	rs80065107	1.39	Europe/North America	*Liu et al.*
	11q24	ETS1	rs12574073	1.33	Japan/China	*Kawashima et al.*
*12*	12p13	TNFRSF1A, LTBR	rs1800693	1.27	Japan/China	*Liu et al.*
	12q24	SH2B3	rs11065979	1.20	Europe/North America	*Liu et al.*
*13*	13q14	TNFSF11	rs3862738	1.33	Europe/North America	*Juran et al., Liu et al.*
*14*	14q24	RAD51B	rs911263	1.26	Europe/North America	*Liu et al.*
	14q32	TNFAIP2	rs8017161	1.22	Europe/North America	*Mells et al.*
*16*	16p12	PRKCB	rs3785396	1.35	Japan/China	*Kawashima et al.*
	16p13	CLEC16yA, SOCS1	rs12708715	1.29	Europe/North America	*Liu et al.*
	16q24	IRF8	rs11117432	1.31	Europe/North America	*Mells et al.*
*17*	17q12	IKZF3	rs17564829	1.26	Europe/North America	*Liu et al.*
	17q12	IKZF3	rs9303277	1.43	Japan/China	*Kawashima et al.*
	17q12	Multiple genes	rs9635726	1.38	Japan/China	*Qui et al.*
	17q21	MAPT	rs17564829	1.25	Europe/North America	*Liu et al.*
*19*	19p12	TYK2	rs34536443	1.91	Europe/North America	*Liu et al.*
	19q13	SPIB	rs3745516	1.46	Europe/North America	*Liu et al.*
*22*	22q13	SYNGR1	rs2267407	1.29	Japan/China	*Liu et al.*

Chr, chromosome; SNP, single-nucleotide polymorphism; OR, odds ratio. Studies are ordered by chromosome.

This review outlines the current evidence about the genetic association between HLA variants and PBC, the link between HLA haplotypes and clinical manifestations in various populations worldwide, the alleged role in disease pathogenesis, and novel therapeutic strategies.

## Genetic studies for HLA variants

From a methodological point of view, it is possible to separate genetics studies regarding HLA in PBC by identifying pre-GWA-, GWA- and post-GWA-studies. A summary of the most significant HLA variants associated with PBC can be found in **Table 2**.

**Table 2:** List of PBC-related HLA associations.

*Reference*	*Year*	*Total subjects (cases+controls)*	*Country*	*HLA associations*
*Uderhill et al.*	1992	321	UK	**DR8**
*Morling et al.*	1992	1227	Denmark	**DR3**
*Gregory et al.*	1993	493	UK	**DR8**
*Onishi et al.*	1994	492	Japan	DRB1*0803
*Begovich et al.*	1994	582	US	DRB1*0801 DRB1*0901
*Donaldson et al.*	2001	266	UK	DRB1*0801 DQA1*0401/DQB1*0402 DRB1*0101/DQA1*0401/DQB1*0402
*Stone et al.*	2002	370	Canada	DRB1*08
*Wassmuth et al.*	2002	256	Sweden	DQB1*0402 DRB*08
*Invernizzi et al.*	2003	707	Italy	DRB*11
*Invernizzi et al.*	2003	670	Italy	B*15 B*41 B*55 B*58
*Mullarkey et al.*	2005	453	US	DQB1*04 DRB1*0801 DQA1*0102 DQB1*0301 DQB1*0602 DRB1*1501
*Donaldson et al.*	2006	411	UK	DQA1*0401
*Donaldson et al.*	2006	175	Italy	DQA1*0401 DQB1*0402 DRB1*08 DQB1*0301 DRB1*11 DRB1*13
*Donaldson et al.*	2006	350	Italy	DRB1*0801/DQA1*0401/DQB1*0402 DRB1*11/DQA1*0501/DQB1*0301 DRB1*13/DQA1*0103/DQB1*0603
*Donaldson et al.*	2006	648	UK	DQB1*0402 DRB1*08 DQB1*0301 DRB1*13
*Donaldson et al.*	2006	1296	UK	DQA1*0401/DQB1*0402 DRB1*0801/DQA1*0401/DQB1*0402
*Invernizzi et al.*	2008	2656	Italy	DRB1*08 DRB1*13
*Nakamura et al.*	2010	1184	Japan	DRB1*0405 DRB1*0803 DRB1*11 DRB1*1101 DRB1*1302 DRB1*1501
*Umemura et al.*	2012	752	Japan	DRB1*0405/DQB1*0401 DRB1*0803/DQB1*0601 DRB1*1101/DQB1*0301 DRB1*1302/DQB1*0604
*Liu et al.*	2012	11275	UK	DQA1*0401 DQB1*0402 DQB1*0302 DRB1*0404 DQB1*0602 DQB1*0301 DRB1*1501 DRB1*1101 DRB1*1104 DQA1*0102 DQA1*0501
*Invernizzi et al.*	2012	2116	US	DRB1*08 DRB1*14 DRB1*11
*Invernizzi et al.*	2012	–	Italy	DRB1*08 DRB1*14 DRB1*11
*Zhao et al.*	2014	645	China	DRB1*0701/DQB1*0202 DRB1*0803/DQB1*0601 DRB1*1202/DQB1*0301
*Almasio et al.*	2016	265	Italy	DRB1*07 DRB1*08
*Clemente et al.*	2017	218	Sardinia	DRB1*0301/DQB1*0201
*Yasunami et al.*	2017	2392	Japan	DRB1*0405/DQB1*0401 DRB1*0803/DQB1*0601 DRB1*1302/DQB1*0604 DRB1*1403/DQB1*0301 Not(DRB1*1403)/DQB1*0301
*Darlay et al.*	2018	11275	UK	DPB1*0301 DPB1*0601 DPB1*1001 DPB1*1701 C*0401 DPA*0201 DPB1*0401
*Li et al.*	2022	–	–	DRB1*0701 DRB1*1401 DRB1*1405

Studies are ordered by year. HLA variants in bold represent predisposing associations, those underlined represent protective ones. Only significant associations (P<0.05) are reported. Approximately, the pre-GWAS era corresponds to years 1992-2009, while GWAS era starts from 2009; post-GWAs technologies have been employed in PBC from years 2021.

### Pre-GWAS era

Before the advent of the GWAS era (approximately from 1992 up to 2009), several candidate studies pointing to the HLA region were performed. The analysis of the HLA region identified a relatively small number of alleles associated with PBC in several independent studies (21–26); these results were more reliable than similar studies on non-HLA alleles, that were mostly underpowered and resulted in several signals not validated in following GWAS (27).

First studies on HLA in PBC were conducted in the early ‘90 on UK population, revealing the presence of *DR8* as predisposing factor for disease onset (21). Donaldson and Invernizzi associated the HLA loci DRB1*08 with an increased risk of PBC in British and Italian individuals respectively, and DRB1*11 and B1*13 with a protective effect in Italians (28, 29). Despite these consistent findings, we should bear in mind that these studies suffered from relatively weak statistical power, a strong potential for type 1 statistical error, and were criticized for their *a priori* approach.

### GWAS era

In GWAS, the frequency of genetic variants (SNPs) is compared between cases and controls. If there is a statistical association sufficiently divergent from the distribution seen in a control group (typically a p-value < 5*10^-8^), variants are considered associated with the phenotype (30). The strength of the association is typically evaluated by the Odds Ratio (OR), calculated based on the allele frequencies in cases and controls. Most of human multifactorial diseases have a complex genetic architecture, with several risk variants contributing little to the disease per se and tagging mostly regulatory regions of the genome.

**Figure 1:**
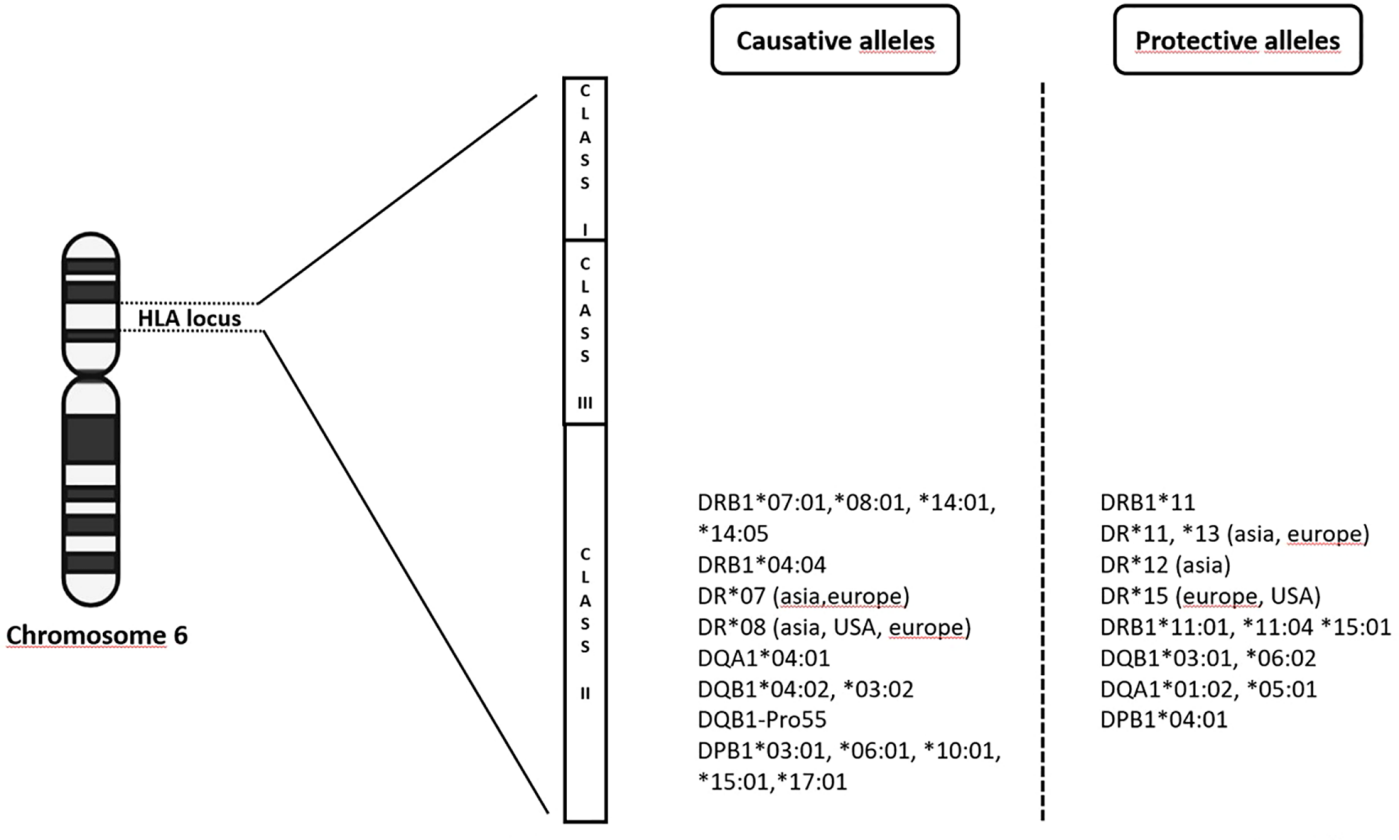
The complex milieux of HLA alleles and its association with PBC (increased vs protective risk).

From 2009 up to the present date, GWAS in PBC have included individuals of European origin (Italy, United Kingdom, USA, Canada) and East Asians (China, Japan), and further analyses, including fine-mapping studies and genome-wide meta-analysis have expanded the list identifying up to 50 genome-wide significant associated variants (12–19, 31–33). Genome wide association studies confirmed that the MHC region remains by far the strongest genetic contributor to PBC susceptibility in terms of OR (**Figure 1**). The HLA-*DRB1* genes (alleles *08, *11, and *14) have been reported for most of the *DRB1* association signal; DRB1*08 is the strongest predisposing allele, whereas DRB1*11 is the protective one (34). A more recent meta-analysis from Li et al. (35) identified HLA DR*07 as risk factor in Asian and European populations, and HLA DR*08 associated with PBC in Asian, American and European subgroups; on the contrary, a protective role of HLA DR*11 and *13 was described in Asian and European populations, while HLA DR*12 decreased risk of PBC in Asian and *15 in European and Americans populations. It is of note that the HLA signals DR*08, DRB1*11, and *13 have been confirmed across different populations, except in Sardinians (36). Furthermore, studies based on UK PBC consortium confirmed the results of the pre-GWAS era. Strong associations were found for HLA-*DQA1* (alleles *04:01), conferring an approximately threefold increased disease risk, HLA-*DQB1* (alleles *04:02, *03:02) and HLA-*DRB1* (alleles *04:04). Conversely, HLA-*DQB1* (alleles *06:02, *03:01), HLA-*DRB1* (alleles *15:01, *11:01, *11:04), HLA-*DQA1* (alleles *01:02, *05:01) have been reported as protective ones (18).

Subsequently, HLA-*DPB1* (alleles *03:01, *06:01, *10:01, *17:01 as causative and alleles *04:01 as protective), HLA-*C* (allele *04:01) and HLA-*DPA* (allele *02:01) were found to be significantly associated within the same cohort (37). In a Chinese cohort, Qiu et al. found 179 SNPs in the MHC region and described the most significant association with the HLA-DRA locus, while a SNP in the HLA-*DRPB1* was the second most significant locus in the MHC region (16).

GWAS allow to associate genetic variants with any phenotype of interest. PBC is typically characterized by the positivity for AMA and/or PBC-specific anti-nuclear antibodies (against the gp210 and sp100 proteins) (38). Positivity for these autoantibodies may occur even before the presence of overt disease and be an isolated laboratory finding. In a Chinese cohort, Wang et al. studied the genetic predisposition to sp100 positivity and identified HLA-DRB1*03:01, DRB1*15:01, DRB1*01 and DPB1*03:01 alleles as the most associated ones (38).

Generally speaking, since association does not mean causation, the region where the leading SNP has been found is usually dissected by the so-called “fine mapping strategy”, that aims to identify the potential biological role of the genetic variant associated with the disease (39). Fine-mapping of the MHC region in Han Chinese court confirmed that HLA-*DRB1* and/or HLA-*DQB1* contributed the strongest signals, and that HLA-*DPB1* was a separate independent locus (40). In addition, the authors performed logistic regression to identify independent associations with amino acid polymorphisms in MHC genes. Three residues (HLA-DRβ1-Ala74, HLA-DQβ1-Pro55 and HLA-DPβ1-Asp84) were found to be independently associated with PBC and all three were located in the peptide-binding groove of MHC II molecules. The 3D protein structure model and electrostatic potentials calculation revealed alternations in antigen-binding affinity leading to an instability of the MHC proteins, that has been proposed as a pathogenetic mechanism in HLA-mediate autoimmune disease (41).

### Post-GWAS era

GWAS have some intrinsic limitations. First of all, GWAS results only account for few percent of the whole genetic risk (the remaining being called “missing heritability”) (42). One reason is that diseases are likely to have some of their genetic risk determined by rare genetic variants that are not included in GWAS (43). Second, GWAS generate hypotheses but require fine-mapping downstream approaches to put their results in the right biological context. Third, many ethnic groups have not been included in GWAS for decades, leaving out a huge number of underserved populations (44). On top of the ethical concerns, when GWAS have been performed on populations with different genetic backgrounds only partial overlap with historical ones has been found, revealing novel variants and generating novel candidates for further dissection.

For all of these reasons, several “post-GWAS” strategies have been developed (45). As far as the study of HLA is concerned, there is mounting interest in studying how genetic alterations result in altered peptides. This is becoming more and more relevant also for clinical purposes, since typing HLA at the amino acid level can tease apart different proteins that can lead to allogeneic responses. For example, as previously described, amino acid alterations located in the antigen-binding site of MHC molecules can lead to instability of the molecule that more likely might form MHC-self-epitope complexes, conferring a risk for autoimmunity (41).

Nonetheless, precise HLA typing remains very challenging due to the high density of polymorphisms in HLA genes, sequence similarity among these genes, and to the extreme level of linkage disequilibrium of the locus (46). High resolution HLA typing is possible with various technologies that are becoming more and more efficient with an increasing reduction in cost. Next-generation sequencing (NGS) methods have been increasingly replacing previous technologies (such as DNA-based typing and sequence-based typing methods) allowing an increasingly precise HLA typing, bringing many advancements to the field of HLA genotyping within relatively few years (47).

Other promising approaches have been under development. For example, the PCR-NGS approach implies the use of standard polymerase chain reaction (PCR) to capture regions of interest, and the resultant amplicons are then subjected to NGS (48). More recently, a new technology called HLAscan has been proposed. HLAscan is a typing multi-step method based on alignment approach, in which short selected sequence reads are aligned with reference HLA alleles obtained from a database and then allele types are determined based on the numbers and distribution patterns of the reads on each reference target. HLAscan demonstrated not only to outperform the established NGS-based methods but also to complete other sequencing-based typing methods (49).

World-wide efforts in developing NGS technologies have dramatically increased the availability of whole-genome sequencing (WGS) and whole-exome sequencing (WES) data. WES and WGS have proved to be effective strategies in discovering rare variants that produce large effects, even in non-monogenic rare disease (50) and in isolated population (51). Li et al. applied WES in three PBC families and associated three new HLA variants (HLA-DRB1*07:01, *14:01 and *14:05) to PBC (52). The authors of this study also describe the amino acid variants located in the peptide-binding site of the MHC molecule and found that DRB1*07:01 was strongly associated with clinical manifestations. Of note, Wang et al. used WES on 90 individuals including 30 PBC probands and identified 19 unreported genes harboring validated *de novo* mutations (DNMGs) (53). Subsequently, the authors interrogated the co-expression dynamics of the DNMGs utilizing a published transcriptome dataset of the CD4^+^ T cells, since they play a critical role in PBC (54). In addition, they found that transcription factor MEF2D and the DNA repair gene PARP2 were highlighted as hub genes and identified to be up- and down-regulated respectively in peripheral blood data. This transcription alteration seems to be the trigger of a series of molecular and cellular processes that have pivotal roles in PBC pathophysiology (53). This represents a small example of the potential of WES/WGS to further improve the understanding of the genetic predisposition to the disease.

## Geoepidemiology

PBC presents different prevalence and incidence rates across populations and geographical areas. Geo-epidemiology has focused on potential environmental and socioeconomic risk factors (55, 56). Studies from the North-East of England have identified a higher prevalence of PBC in urban areas that have a strong coal-mining heritage (55), whereas a study in New York City found clusters of PBC patients in zip codes that contained, or were adjacent to, superfund toxic waste sites (56).

Other environmental factors associated with increased risk of PBC were cigarette smoking, hair dyes, the use of hormone replacement therapies, and recurrent urinary tract infections (UTI) (3). As it concerns the latter molecular mimicry analysis has shown that PBC autoantigene pyruvate dehydrogenase complex E2 subunit (PDC‐E2) has common structure with the Escherichia coli E2 subunit of the 2‐oxo‐acid dehydrogenase complexes (57). This suggested that the infection with E. coli, a predominant pathogen in most cases with UTI, is a key factor in breaking immunological tolerance in PBC (58).

In addition to environmental factors, there is known variation in allele frequencies among different ethnic groups. Unfortunately, PBC GWAS have mostly been performed on subjects of European ancestry and more recently on east-Asians (Han Chinese and Japanese). At the time of writing, genetic studies on “neglected population” such as African, Latin American or Australian population are still lacking.

## The role of HLA in PBC pathogenesis: Imperfect HLA interactions

The most recent model of PBC pathogenesis comprises two phases: an initiation phase, characterized by autoimmune phenomena driven by defects in biliary homeostasis and aberrant antigen presentation; and a progression phase, driven by the retention of bile acids (2). Based on this model, one would expect that HLA would play its major pathogenetic role at the initiation phase. Despite the association between HLA and autoimmune diseases have been known since 1970s (59), the exact pathogenic mechanism that links HLA and disease onset remains largely elusive (60). The scientific progress in genetic and molecular profiling technologies represents a big hope for the future, since it is gradually unveiling the complex interaction between HLA, peptides and TCR (61). Several structural and associated functional studies were conducted, with more than 50 unique TCR-peptide-HLA structures recognized (62).

**Figure 2:**
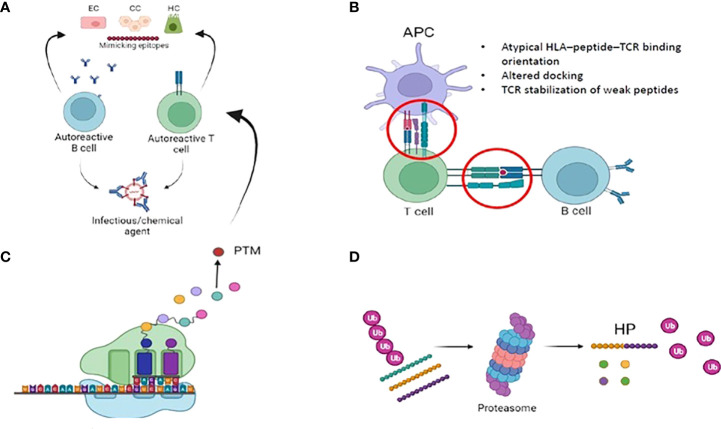
Imperfect HLA interactions and autoimmunity: **(A)** Molecular mimicry: Foreign antigens (i.e. either from infecious or chemical agents) with similarities to self-antigens are primed by T and B cells, that autoreactive and trigger autoimmunity. **(B)** Immunological synapse and autoimmunity: several mechanisms of the complex interplay between APC, B- and T- cells might lead to the activation of autoimmune B and T cells. Among them, mechanical binding alterations (atypical binding orientation, altered docking) and TCR stabilization of weak peptide. Weak HLA binding of self-peptides might contribute to autoimmunity by allowing the escape of autoreactive T-cellsfrom the thymus. **(C)** Post translational modifications (PTM): they are spontaneous or enzymatically induced modifications of one or more amino acids occuring after protein biosynthesis. After PTM, these proteins become modified self-antigens, and they do not “tolerize” developing thymocytes. Consequently, modified self-antigens can be taken and processed by APC, that will present them to autoreacitve T and B cells. **(D)**: Generation of hybrid peptides: proteasomal-mediated degradation and splicing of intracellular self-peptides can promote the generation of non-self-hybrid peptides. This mechanism occurs with high protein concentrations in a confined environment, that favours protease-mediated peptide fusion.

A variety of physiologic and steric factors can affect the TCR-peptide-HLA interface (61). Further, several mechanisms of imperfect HLA interactions were identified as potential disease triggers in several autoimmune disorders. They include: i) molecular mimicry and mechanical binding alterations (HLA–peptide–TCR binding orientation, low-affinity peptide binding), ii) post-translational epitope modification or iii) generation of hybrid peptides (63) (**Figure 2**). As for the latter, no data are available in PBC. We will thus briefly focus on the first two mechanisms.

### Molecular mimicry and mechanical binding alterations

Molecular mimicry is a well-accepted mechanism linking infections and autoimmunity onset. It consists in the antibody- and cellular-mediated immune response against self-protein(s) sharing a sufficient degree of similarity with epitopes of infectious agents (64). Almost all patients with PBC have autoantibodies recognizing the E2 subunit of the pyruvate dehydrogenase complex (PDC-E2) or other related mitochondrial enzymes complexes. Thus, cross reactivity should not be a surprise since such mitochondrial enzymes have conserved sequences shared across different species (65). Shimoda et al. nicely demonstrated the mechanism of molecular mimicry of AMA and antinuclear autoantibodies in PBC, and also showed that the mimicry peptide can become the immunodominant T-cell epitope (57, 66). Different bacterial strains and xenobiotics were considered as possible causative of cross reaction in PBC. Among them, E. coli and *Novosphingobium aromaticivorans* are considered among the best candidates for the induction of PBC (67–71).

Along with molecular mimicry, mechanical binding alterations were considered among the triggers of autoimmune disorders (63). Somatic hypermutation (SHM) is a process occurring within germinal centres, aimed at producing high-affinity antibodies *via* the introduction of somatic mutations in the rearranged region of the Ig genes. Activation-induced cytidine deaminase (AID) is required for SHM to occur, and it leads to DNA mutations by conversion of cytosine into uracil (72). While some studies showed that AID can contribute to the loss of self-reactivity in mice and humans (73–76), other have attributed a role of AID and SHM deficiencies in autoimmune disease onset and severity (77–81). As for PBC, a single work assessed a limited SHMs in class-switched isotypes in PBC patients, which may be indicative of low-affinity Ag binding (82). To the best of our knowledge, data on HLA–peptide–TCR binding orientation in PBC are still lacking.

### Post-translational epitope modification

Post-translational modification refers to the addition of a functional group to a protein or to the proteolytic processing and folding necessary its functional maturation. Acetylation, methylation, phosphorylation, ubiquitination and sumoylation are among the most common functional group additions. These modifications have fundamental roles in chromatin structure and function.

Reduced methylation levels of the cluster of differentiation 40 ligand (CD40L) promoter were described in peripheral CD4 T cells in PBC patients, and this correlated to increased immunoglobulin M (IgM) serum concentration, a typical serological finding of PBC (83). An increased variety of methylation patterns were on chromosome X of a small cohort of monozygotic twins with PBC (84). A scan of the X chromosome revealed a hypomethylation of the promoter of a C-X-C motif chemokine receptor 3 (CXCR3) (85). CXCR3 plays a key role in dysimmunity, and increased levels of this receptor were described among PBC patients (86).

T cells from PBC patients showed an increased concentration of β-arrestin 1, a protein involved in CD4+ T cells histone acetylation, and its overexpression correlated to hepatic disease severity (87). Upregulated βarr1 in PBC associated to decreased levels of TNF-related apoptosis-inducing ligand (TRAIL), involved in autoimmunity prevention through cell cycle arrest (87). The role of non-coding RNAs (ncRNAs) was largely studied in PBC. They are RNAs not translated into proteins. Scientific focus was directed towards microRNAs (miRNAs), a specific class of ncRNA with the ability to regulate gene expression (88). Among miRNAs, MiR-506 overexpression was described in PBC. This specific miRNA targets the anion exchanger 2 (AE2), an important protein involved in biliary epithelium homeostasis (89). Of note, miR-506 targeted cholangiocytes showed an higher expression of pro-inflammatory and pro-fibrotic markers (90). A novel and interesting topic in PBC is represented by the connection between epigenetic alterations and X chromosome monosomy/inactivation abnormalities (91). Deep investigation on the impact of epigenetic modifications in X chromosome alterations in PBC might reveal important insights on disease specific sex imbalance and pathogenesis.

## The role of biliary epithelial cells in PBC pathogenesis

The recognition of self-peptides selectively presented by susceptible HLA has gained scientific interest for a long time. Specific HLA loci, when combined with certain molecules, may confer either protection or susceptibility to develop autoimmunity. In rheumatoid arthritis, variants at 71β position of certain HLA haplotypes are responsible for the presentation of citrullinated peptides, recognized as non-self by T cells (92). Similarly, the presentation of the reduced form of insulin to specific DRB1 protein haplotypes correlated to insulin autoimmune syndrome (93). As for PBC interesting data are emerging on the role of biliary epithelial cells [BECs, aka cholangiocytes (CC)] as possible triggers of autoimmunity. Cholangiocytes line the biliary tree from the small intrahepatic Hering canals to the large extrahepatic bile ducts (94). They are endowed with the ability to regulate bile production, fluidity, and homeostasis (95). Evidence showed that CC actively participate in the innate and adaptive immune responses, acting as innate immune cells, antigen presenting cells, and expressing cytokines, chemokines, and adhesion molecules (96). In this way, they protect hepatocytes from potentially toxic bile acids, microbial products, and the direct translocation of gut-derived microbes (97). BECs constitutively express HLA class I associated to CD8 cytotoxic activity, as it occurs for all mammalian epithelial cells. However, differently from other epithelial cells, they are capable of antigen-uptake and can upregulate HLA class II peptides in response to the local release of inflammatory cytokines, in particular IFN-γ and IL-2 (98).

This process was described in several autoimmune cholangiopathies, including PBC (99–102). Despite this, several studies failed to demonstrate that HLA class II expressed by BECs can directly present peptide antigens to T cells (54, 101, 103). This might occur secondary to the lack of B7-1 (CD80) and B7-2 (CD86) co-stimulatory ligands, required for T cell activation. The inefficient peptide antigen presentation, together with the expression of inhibitory molecule such as PDL1 and PDL2, result in T-cell anergy. This suggested a possible role of BECs as accessory cells rather than proper antigen presenting cells, at least for peptide antigen presentation (104–106).

A breakthrough in the recent history of immunology was the discovery of CD1-restricted T cells, capable of a subset of HLA I-like molecules with the ability to present self and microbial lipid antigens to T cells (107). The scientific excitement associated with this discovery grew dramatically with the recognition of CD1d-restricted NKT cells, a subset of T-lymphocytes bridging innate and adaptive immunity (108). Most NKT can recognize self-lipid antigens, but the antigen capability to activate NKT and trigger cytokine release depends largely on TCR signalling strength and the presence of co-stimulatory signals (109). NKT can rapidly express pro- and anti-inflammatory cytokines and are endowed with split roles in several pathologic conditions, including autoimmune disorders and malignancies. They can inhibit or exacerbate autoimmunity, and this feature makes them an interesting target for treatment (110, 111).

Cholangiocytes can present lipid antigens through CD1d and the HLA-related receptor MR1, respectively (112–114). In particular, MR1 activates a specific subset of T cells, the mucosal associated invariant T cells (MAIT), that are located in the peribiliary sinusoids close to the portal tracts, thus being in straight contact with gut bacteria antigens. Peribiliary MAIT localization was described in several hepatic disorders including PBC, autoimmune hepatitis and primary sclerosing cholangitis (115).

As for PBC, MAIT cells seem to have a protective, since their levels is reduced as compared to healthy livers (116).

CD1d is upregulated on biliary epithelial cells during the early stages of PBC (113). *In vitro* models showed the ability of CD1d to activate natural killer T cells through the presentation of endogenous and microbiome-derived lipid antigens (112). Whether the expression of HLA class II molecules might represent an epiphenomenon of BECs activation, or it might link cholangiocytes and autoimmunity is unknown, and further studies are awaited.

## The strength of unity in autoimmunity: Shared HLA recognition

Specific HLA subtypes are shared among different autoimmune disorders (117). This feature has attracted the scientific interest in the search for molecular mechanisms behind this correlation (63). Conversely, several MHC−I-pathies share immunopathogenic basis despite different HLA genotypes (118). The ability to compare HLA commonalities and differences among autoimmune diseases can translate into the recognition of shared or distinct pathophysiological pathways, thus increasing the understanding of disease aetiology and therapy. As for HLA class II, different allele interactions were identified for multiple sclerosis, rheumatoid arthritis, type 1 diabetes, and coeliac disease (119). However, large data collection and sharing enabled by technological advances will ease the process of common disease pattern recognition.

## The role of HLA-mediated antigen recognition

The pool of endogenous and exogenous antigens presented by each HLA molecule varies enormously not only among different subjects but also within different tissues of the same subject. The rapid pace of epitope recognition fostered by novel quantitative mass spectrometry technologies enabled the recognition of millions of peptide repertoires, collected from blood and target tissue samples (120). In chronic autoimmune disorders, epitope heterogeneity coupled with HLA polymorphisms leads to a continuous competition of T cells for the most immunodominant epitopes. This resulted in the development of a large T cell clonotypes repertoire, consequence of lifetime modifications occurring from the early thymic T-cell development to specific events occurring in peripheral tissues (121). In liver tissues derived from PBC patients, disease-associated clonotypes were detected irrespectively of the presence of DRB1*08:01/DRB1*04:04 susceptibility alleles, with three clonotypes detected in 40% of the cases. Further, antigen-driven clonal selection and expansion was evidenced, with HLA restricted autoreactive T cells specific for the PDC-E2 (122). In addition, T-cell repertoires differ among autoimmune liver diseases (123), and this observation led to the concept of disease-specific imprinting from antigenic repertoires.

To conclude, the recognition of PBC-specific HLA susceptibility can translate into specific T-cell repertoires. This might consequently lead to the recognition and selective targeting of disease-specific T-cells, with possible treatment implications for the future.

## The imperfect HLA-antigen-TCR binding behind autoimmunity

It has been long recognized that the strength of HLA-peptide-TCR interaction can influence the thymic fate of a TCR (124). In particular, weaker interactions promote positive selection and T cell survival, while stronger ones drive clonal deletion and differentiation into Treg phenotype.

The study of highly polymorphic TCR sequences and HLA alleles was enabled only in recent years, thanks to technological advances in deep-sequencing technologies (125). TCR receptors consist of an α- and a β-chain. They include three highly variable peptide loops, each encoded by a specific gene, that protrude toward the pMHC complex. Of them, the complementarity-determining region (CDR3β) in the β chain is the most variable, and it mediates antigen recognition (126). Hydrophobicity at specific positions of the β−chain CDR3β was demonstrated to promote reactivity to self-peptides (127, 128). Recent evidence highlighted the role of TCR β variable gene (TRBV), encoding for a region of CDR3β, in the mechanism of autoimmunity. In particular, TRBV shapes the TCR affinity to conserved HLA sites, thus altering their propensity towards self vs non-self-activation (129). A study of peptide-HLA libraries coupled to deep-sequencing analysis attested a higher tolerance for substitutions to peptide residues that are located distally to the TCR-binding site, and some of them were identified through computational analysis (130). The TCR-peptide-HLA docking mechanism is not always perfect, and engagement of homologous antigens *via* distinct docking geometries was described. TCR-antigen cross reaction might lead to the breakdown of immune tolerance, potentially triggering autoimmunity (131, 132). A recent study used NGS to characterize and compare the TCRβ chain of peripheral T cells in PBC patients vs controls (68). Results showed that PBC patients carried TCRβ with reduced CDR3 length and that TCRβs bearing shorter CDR3 loops, which are associated with abnormal insertion length at the variable, diversity and joining gene segments in the β-chain locus. The functional outcome of this structural alteration is yet to be demonstrated.

## Exploiting the HLA-peptide-TCR binding to modulate autoimmunity: Antigen specific immunotolerance

Antigen-specific immunotolerance (ASI) represents one of the most promising therapeutic approaches for autoimmune disorders. It has been long practiced in the field of allergy (133, 134), and it consists in the promotion of antigenic specific tolerance by inhibiting autoantigen-specific immune response, while sparing the rest of the immune system. In fact, despite the revolutionary potential of immune suppressive treatments in autoimmune and inflammatory disorders, those are predominantly symptomatic approaches not acting on disease cause and associating to several side effects. The inhibition of autoantigen-specific immune response though ASI represents a clever, innovative, and safer approach. It mainly relies on the administration of antigenic epitopes derived from the same proteins targeted by the autoreactive lymphocytes. Prior to antigen administration, specific antigenic conditioning is required to induce a regulatory response capable to restore homeostasis and to aid the deletion of autoreactive cones. The HLA background has a strong influence on the efficacy of ASI, since HLA heterogeneity largely shapes the antigen-specific immune responses. Individual HLA background should be considered in clinical trials exploiting ASI, and early tolerization should be encouraged to reduce the likelihood of epitope spreading.

**Figure 3:**
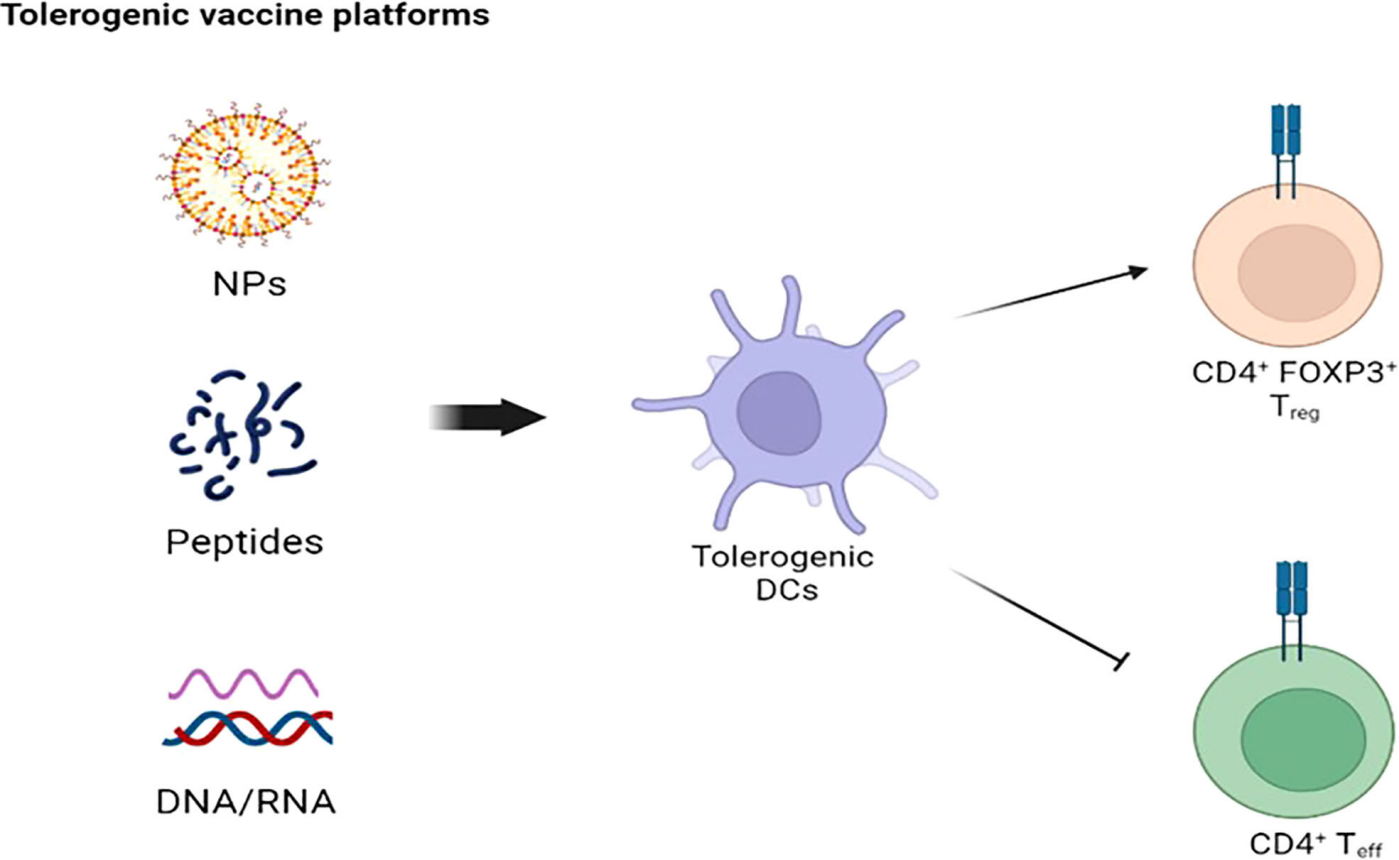
Tolerogenic vaccine platforms: The goal of NPs-, peptides-, and DNA/RNA-based platformes is to stimulate tolerogenic DCs to suppress CD4^+^ T cell-mediated autoimmunity. NPS, nanoparticles; DC, dendritic cells.

The use of ASI recently increased also in autoimmune disorders, after a preliminary success in experimental models (135). So far, ASI focused on antigen presenting cells (APC), in particular dendritic cells (DC), and CD4 T cells, responsible for the onset and perpetuation of most autoimmune-related processes (136). Tolerogenic vaccines represent the mainstay of ASI, and several vaccines platforms were built throughout the years (**Figure 3**). Since APC represent the main actors of antigen specific tolerance, strong efforts were directed towards the recognition and delivering of autoantigens to specific APC subtypes (136–138). There are several mechanisms of action behind the induction of antigen-specific tolerance, each with a specific antigen delivery approach (139). A major limit of ASI in most autoimmune disorders is the poor knowledge of the whole autoantigen panel involved and the uncertainty of whether tolerance towards the recognized autoantigen/s is enough to reverse autoimmunity, since several autoimmune disorders have autoantibodies associated with disease but without a role in disease pathogenesis (140). ASI should rely on antigens capable of modulating CD4 T cells without engaging pathogenic B or T cells. In this way, a correct balance between efficacy and safety is guaranteed.

Among the different antigen delivery approaches, the use of nanoparticles seems promising, with great interest for tolerogenic nanoparticles (tNPs). A particular subtype of tNPs, called tolerogenic immune modifying nanoparticles (TIMP) were designed to achieve antigen-specific and site-specific immune tolerance (141).

TIMP use has steeply increased, emerging from cell-based to synthetic antigen carriers and from animal models to clinical trials. In particular, the role of TIMP was studied in some autoimmune disorders, such as experimental autoimmune encephalomyelitis (the mouse model for multiple sclerosis), type 1 diabetes, systemic lupus erythematosus and multiple sclerosis (142–149). Of note, up to 99% of nanoparticles pass through the liver, and most of them are taken up and internalized by scavenger receptors of hepatic Kupffer cells (150, 151).

The tolerogenic nature of liver fosters the induction of antigen-specific regulatory T cells (Tregs), a subset of CD4+ T cells with a critical role in maintaining immune system homeostasis and immune tolerance. The contact between nanoparticles carrying autoantigens and Tregs favours the induction and activation of latter (152). PBC represents an ideal target disease for nanotechnology, since autoantibodies are disease- and organ-specific, thus increasing the specificity of nanoparticles-delivery. The major actors of PBC immunopathogenesis are AMA, T cells and biliary epithelial cells, while the inner lipoyl domain of PDC-E2 is the dominant autoepitope (153). The recognition of the correct timing and dosing of antigen delivered by nanoparticles are limiting steps in the process of immunogenicity, and research is ongoing at an exciting pace. TIMP is a potentially safe approach, and it represents a potential shift from immunosuppressive to tolerance inducing therapies in autoimmune disorders (154–156). As mentioned above, Tregs orchestrate the immune system homeostasis. They express high levels of transcription factor Forkhead box protein P3 (FOXP3), critical for their thymic development and activity (157). FOXP3+ Tregs exert a suppressive activity for autoreactive T and B cells (158), and its absence results in severe autoimmune and immune-related disorders (159, 160). Treg dysfunction might be responsible for the uncontrolled T cell reaction against cholangiocytes, as showed by Jeffrey et al. (161). PBC patients showed a reduced level of peripheral Tregs (162, 163), and the silencing or reduced activity of FOXP3 in murine models led to peri-biliary immune damage and AMA production (164, 165). Of interest, reduced levels of Tregs were recorders also in the peripheral blood of daughters and sisters of PBC patients (162, 163). The expansion of Tregs represents a promising treatment approach in autoimmune disorders, even if several barriers need to be overcome to achieve a high quality Treg cell therapy. Among them, mentions should be done to the difficulty of Treg expansion and the lack of Treg-exclusive markers (136).

## Conclusions

PBC is a complex autoimmune disorder with high inter- and intra- individual variability. HLA has long been studied in PBC, and associations were first described in the pre-GWAS era and largely expanded thanks to the rapid spread of GWAS platforms. However, such platforms are not informative on the causal genes or the disease mechanisms, explain only a small fraction of the missing heritability, do not allow to assess rare disease variants, and were not implemented for several populations worldwide. The rapid pace of technological innovation, with the development of novel sophisticated sequencing technologies will gradually unveiling the “hidden” genomic signatures, possibly enabling a complete disease HLA (and non-) mapping. The role of HLA in PBC, as it occurs for most autoimmune disorders, is still to be determined and detailed. The HLA-peptide-TCR interaction has been the focus for long time, but we are far from an exhaustive explanation on how it might be involved in autoimmunity. While PBC pathogenesis remains far from being determined, there is a rapid and constant surge of novel treatment approaches aimed at limiting the autoimmune clones to guarantee antigen specific immunotolerance. Among them, scientific excitement surrounds the discovery of tolerogenic vaccine platforms aimed at the expansion of Tregs.

## Author contributions

GM and AP equally searched the literature and contributed to the first draft of the manuscript. All authors provided critical feedbacks and helped to shape the research, analysis and manuscript. PI, MEG, RA and AG reviewed for important intellectual contents. All the authors approved the final version of the manuscript.

## Funding

This research was partially supported by the Italian Ministry of University and Research (MIUR)—Department of Excellence project PREMIA (PREcision MedIcine Approach: bringing biomarker research to clinic).

## Acknowledgments

The authors thank AMAF Monza ONLUS and AIRCS for the unrestricted research funding.

## Conflict of interest

The authors declare that the research was conducted in the absence of any commercial or financial relationships that could be construed as a potential conflict of interest.

## Publisher’s note

All claims expressed in this article are solely those of the authors and do not necessarily represent those of their affiliated organizations, or those of the publisher, the editors and the reviewers. Any product that may be evaluated in this article, or claim that may be made by its manufacturer, is not guaranteed or endorsed by the publisher.
